# Perceptual uncertainty and action consequences independently affect hand movements in a virtual environment

**DOI:** 10.1038/s41598-020-78378-z

**Published:** 2020-12-18

**Authors:** Martin Giesel, Anna Nowakowska, Julie M. Harris, Constanze Hesse

**Affiliations:** 1 School of Psychology, University of Aberdeen, Aberdeen, AB24 3FX UK; 2 School of Psychology and Neuroscience, University of St Andrews, St Andrews, KY16 9JP UK

**Keywords:** Human behaviour, Psychology, Neuroscience, Sensorimotor processing, Decision

## Abstract

When we use virtual and augmented reality (VR/AR) environments to investigate behaviour or train motor skills, we expect that the insights or skills acquired in VR/AR transfer to real-world settings. Motor behaviour is strongly influenced by perceptual uncertainty and the expected consequences of actions. VR/AR differ in both of these aspects from natural environments. Perceptual information in VR/AR is less reliable than in natural environments, and the knowledge of acting in a virtual environment might modulate our expectations of action consequences. Using mirror reflections to create a virtual environment free of perceptual artefacts, we show that hand movements in an obstacle avoidance task systematically differed between real and virtual obstacles and that these behavioural differences occurred independent of the quality of the available perceptual information. This suggests that even when perceptual correspondence between natural and virtual environments is achieved, action correspondence does not necessarily follow due to the disparity in the expected consequences of actions in the two environments.

## Introduction

While there is little doubt about the potential of virtual (VR) and augmented (AR) reality devices for research and training purposes, it is not clear to what degree virtual environments currently provide a good representation of natural environments^1–3^ and how much correspondence between virtual and natural environments can theoretically be achieved. The underlying problem extends beyond the question of the perceptual quality (fidelity) of simulated environments. If we want actions in VR/AR to be an adequate model for actions in natural environments, we must ensure that the same sources of information that influence the planning and execution of movements in natural environments are also available in VR/AR^4^. While previous research has focussed on the effects of perceptual uncertainty resulting from missing or inadequate implementations of perceptual cues (e.g., binocular cues to depth), the role of higher level factors (e.g., expectations of action related consequences) in virtual environments has so far been investigated to a much lesser extent^1^. Research on sensorimotor decision making has shown that motor behaviour is strongly influenced by perceptual uncertainty and the expected consequences of actions, i.e., gains and/or costs (for reviews see^5,6^). Here, we study these two factors, perceptual uncertainty and expected consequences of actions, jointly, and directly measure the effects of acting in virtual environments on visually-guided hand movements.

We manipulated perceptual uncertainty and expected consequences of actions during an obstacle avoidance task. In such a task, both of these manipulations influence the chances of a collision with the obstacles in a given condition, a potentially dangerous consequence of the action. Higher perceptual uncertainty results when there is less (or less reliable) perceptual information and makes it more difficult to accurately perceive action-relevant features, i.e., in our case the height of the obstacles. We manipulated perceptual uncertainty here by presenting the obstacles binocularly (low uncertainty) or monocularly (high uncertainty), because estimates of size and distance have been found to be less accurate in monocular than in binocular viewing^7–9^ due to the absence of binocular cues to depth. Higher perceptual uncertainty is expected to increase the probability of making an error during movement execution and has been shown to result in a larger opening of the hand (maximum-grip-aperture) in grasping tasks^10–14^ and larger passing distances in obstacle avoidance tasks^15^.

The pressure to perform actions accurately increases with the expectation of negative consequences (costs) resulting from movement errors (e.g., bumping into an object). Higher error-related costs have been shown to increase the distance participants keep from penalty regions when performing targeted pointing movements^16,17^. When higher error-related costs were implied by changes in obstacle properties (e.g., empty or filled glasses), participants kept a larger distance from these obstacles in an obstacle avoidance task^18^. Here, using mirror reflections, rather than computer-generated VR/AR environments (that often contain perceptual artefacts), we created a virtual environment that allowed us to vary the costs of potential movement errors without changing the perceptual information or the appearance of the obstacles^19^ and, thus, to measure the isolated effects of action consequences on avoidance behaviour.

## Results

**Figure 1:**
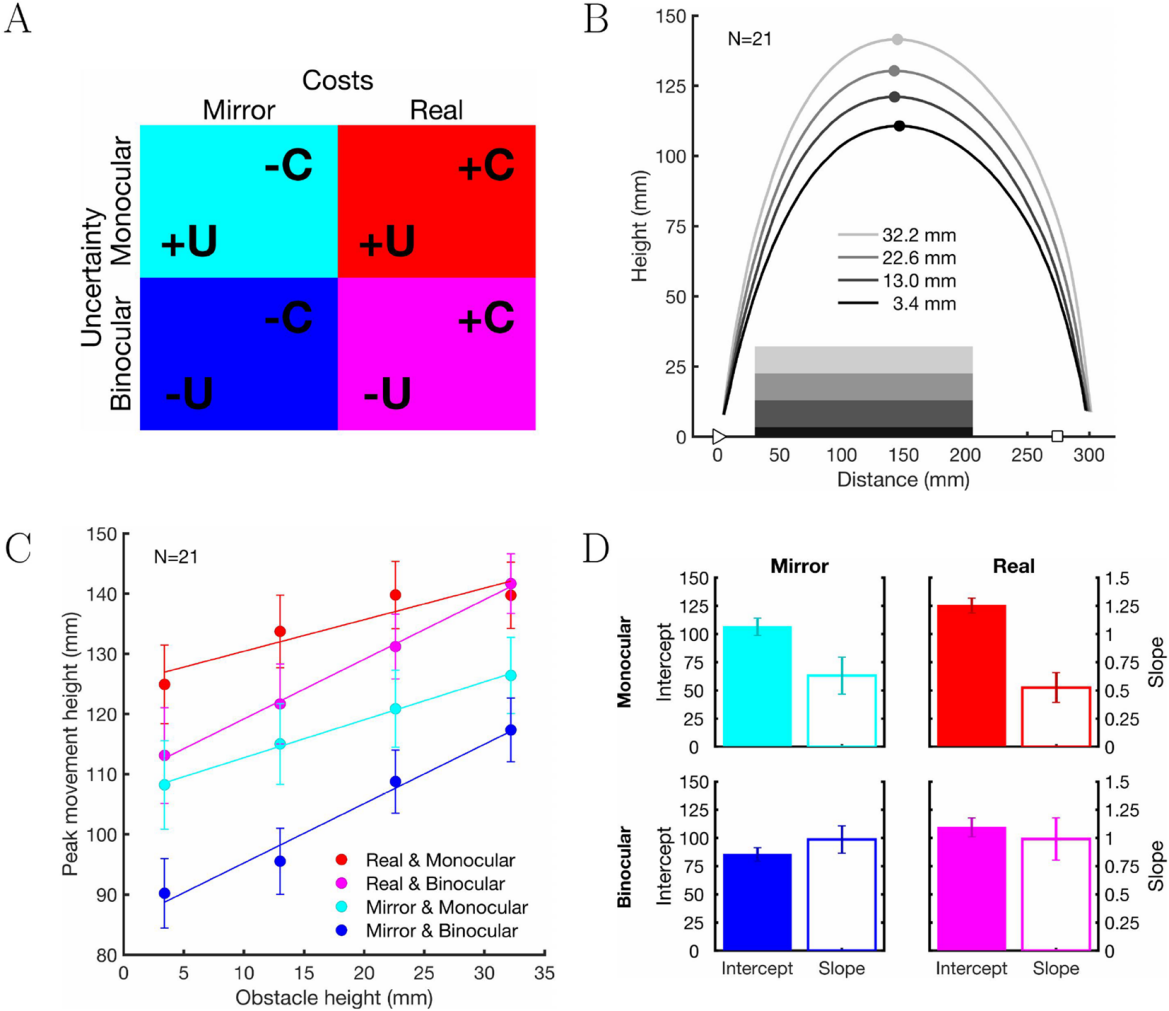
(**A**) Four experimental conditions: perceptual uncertainty is shown in rows and costs of mistakes are shown in columns. U represents the magnitude of perceptual (visual) uncertainty (+U: higher uncertainty, –U: lower uncertainty), and C represents the magnitude of costs of mistakes during movement execution (+C: higher cost, –C: lower costs). (**B**) Example hand movement trajectories for the *real* & *binocular* condition normalized and averaged over participants separately for the four different obstacle heights (3.4, 13.0, 22.6, and 32.2 mm). The x-axis shows the distance from the start position (triangle) to the target position (square) in millimetres. The y-axis shows the height of the movements (mm). The grey-shaded horizontal bars indicate the height and extent of the different obstacles. Curves show the averaged trajectories for the different obstacle heights as indicated by the grey level. The filled circles indicate the peak height of the trajectories (mm). (**C**) Peak height (mm) averaged over 21 participants separately for the four experimental conditions. (red: *real* & *monocular*, magenta: *real* & *binocular*, cyan: *mirror* & *monocular*, blue: *mirror* & *binocular*). The x-axis shows the height of the obstacles (mm) and the y-axis shows the peak height of the reaching movements (mm). Error bars show ±1 SEM. Straight lines show linear regression lines. (**D**) Mean intercepts (baseline safety margin) and slopes (sensitivity to changes in obstacle height) for each of the four combinations of experimental conditions (as shown in **A**). Baseline safety margins were largest for the *monocular* & *real* condition (red), and lowest for the *binocular* & *mirror* condition (blue). Error bars show ±1 SEM.

In an obstacle avoidance task, we measured the trajectories of participants’ right hand moving over obstacles of four different heights (3.4, 13.0, 22.6, and 32.2 mm). Participants performed the movement without visual feedback (open-loop), i.e., their vision was occluded as soon as the hand started to move (see Methods for details). Figure 1A shows how perceptual uncertainty and the expected costs were systematically varied in the different experimental conditions. *Monocular* and *binocular* viewing conditions gave us high (+U) and low (–U) perceptual uncertainty, respectively. The *real* and *mirror* conditions allowed us to manipulate the expected costs of mistakes during movement execution, high costs (+C) and low costs (–C), respectively.

Based on previous research, we predict that the most ’dangerous condition’ should be *monocular* & *real* (top right in Fig. 1A) where both perceptual uncertainty and costs of mistakes are high (+U & +C). Dangerousness should be lowest when perceptual uncertainty is low and no costs of mistakes are to be expected (–U & –C, bottom left in Fig. 1A), i.e., in the *binocular* & *mirror* condition. For the two remaining conditions, *binocular* & *real* (–U & +C) and *monocular* & *mirror* (+U & –C), we expect ’dangerousness’ to be in between the two extreme conditions.

We measured participants’ hand trajectories when reaching from a start point over the obstacles to the target position (see Fig. 1B). For each trajectory, we determined the peak height of the hand, i.e., the maximal vertical displacement of the hand during the movement. Figure 1C shows the peak height of the hand averaged over participants for the four experimental conditions. We analysed the peak height data using a 2 $\times$ 2 $\times$ 4 repeated-measures ANOVA with the factors *costs* (real vs mirror), *perceptual uncertainty* (binocular vs monocular) and *obstacle height* (3.4, 13.0, 22.6, and 32.2 mm). We found that all three factors significantly affected the peak movement height: *costs* ($F(1,20)=43.643$, ${p}<.001$, $\eta _p^2=0.686$), *perceptual uncertainty* ($F(1,20)=23.032$, ${p}<.001$, $\eta _p^2=0.535$), and *obstacle height* ($F(1.234,24.679)=35.026$, ${p}<.001$, $\eta _p^2=0.637$). The interaction between *perceptual uncertainty* $\times$ *obstacle height* was statistically significant ($F(2.433,48.666)=11.378$, $p<.001$, $\eta _p^2=0.363$). Results for all other interactions were not significant (all ${p}\ge .202$, $\eta _p^2\le 0.080$).

We also analysed two other aspects of the peak height data. First, the rate of change in peak height informs us how sensitive the motor system is to changes in obstacle height and thus provides a measure for perceptual accuracy (given that motor uncertainty remains constant). This can be measured as the slope of the peak height data in Fig. 1C. In this case, a slope of 1 would indicate that an increase in obstacle height by, e.g., 10 mm would result in an increase of the hand’s peak height of 10 mm, too, meaning that the hand is kept at a constant distance from all obstacles. In contrast, a slope of 0 would mean that the hand’s peak height does not change at all with increases in obstacle height. Second, the height of the hand over the ground surface provides an estimate of the inferred dangerousness of a condition resulting from a particular combination of perceptual uncertainty and expected costs. We will refer to the height offset that is common to all obstacles (independent of their height) in a particular condition as the baseline safety margin. It can be measured as the intercept of the peak height data in Fig. 1C. We derived slopes and intercepts by computing linear regression fits to the peak height data for each condition. The lines in Fig. 1C show regression lines based on the averaged regression parameters resulting from fits to individual participants’ data.

Figure 1D (open bars, see also Table S1 in the Supplementary Information) shows that the averaged slopes were close to one for binocular viewing and roughly halved in magnitude for monocular viewing. A 2 $\times$ 2 repeated-measures ANOVA with the factors *costs* and *perceptual uncertainty* showed that sensitivity to changes in obstacle height was significantly affected by *perceptual uncertainty* (${F}(1,20)=17.744$, ${p}<.001$, $\eta _p^2=0.470$), with slopes being steeper for binocular (less uncertainty) than for monocular viewing (more uncertainty). There was no effect of *costs* on the slopes (${F}(1,20)=0.211$, ${p}=.651$, $\eta _p^2=0.010$). The interaction between both factors was not statistically significant (${F}(1,20)=0.326$, ${p}=.574$, $\eta _p^2=0.016$). The finding that slopes were shallower when perceptual uncertainty increased is consistent with the underestimation of sizes and distances under monocular viewing, as reported in size and distance estimation experiments^7–9^. Furthermore, the similarity of the slopes in the *real* and the *mirror* conditions indicates that sensitivity to changes in height was similar in these conditions. These results are in line with those from size and distance estimation experiments comparing estimations for real and mirrored objects^19^, and further confirm that mirrors provide complete and reliable visual information.

Figure 1D (filled bars, see also Table S2 in the Supplementary Information) shows that the averaged baseline safety margins, as measured by the intercepts of the lines shown in Fig. 1C, increased with *perceptual uncertainty* (larger for monocular than for binocular) as well as with the costs of errors (larger for real than for mirror). The intercept was largest for the *monocular* & *real* condition, and it was smallest for the *binocular* & *mirror* condition. The intercepts for the two remaining conditions (*monocular* & *mirror* and *binocular* & *real*) were similar and in between the two other conditions. This order of intercepts, and thus baseline safety margins, exactly reflects the predicted order of ’dangerousness’ for the different conditions based on the different combinations of perceptual uncertainty and error-related costs. A 2 $\times$ 2 repeated-measures ANOVA with the factors *costs* and *perceptual uncertainty* showed that both main effects were highly statistically significant (*costs*: ${F}(1,20)=31.243$, ${p}<.001$, $\eta _p^2=0.610$; *perceptual uncertainty*: ${F}(1,20)=45.866$, ${p}<.001$, $\eta _p^2=0.696$). There was no statistically significant interaction (${F}(1,20)=0.443$, $p=.513$, $\eta _p^2=0.022$) between the two factors suggesting that the effects of perceptual uncertainty and costs on safety margins are independent of each other.

## Discussion

In the following, we will first discuss how these findings advance our knowledge about sensorimotor control and sensorimotor decision making, and then elaborate on what they imply for the use of VR/AR as a tool for investigating or training motor behaviour.

### Implications for sensorimotor control

In grasping, increased perceptual uncertainty about the true size and position of a target seems to reliably cause an overall increase in maximum-grip-aperture, i.e., the largest distance between thumb and index finger when approaching an object^10,11,20,21^. Specifically, the effects that the removal of binocular cues has on hand movements have been relatively well studied in this context^10,13,22,23^. As grasping movements are very finely tuned to object size^24,25^, which is underestimated in monocular viewing, one could expect that grip apertures decrease when grasping objects monocularly. While decreased grip apertures were observed by Servos et al.^23^, subsequent studies have consistently revealed larger apertures in monocular viewing conditions^10,12,13,22^. A convincing explanation for this seeming paradox was provided by Jackson et al.^10^ who found that, consistent with the expected underestimation of object size, anticipatory grip forces decreased in monocular grasping while grip apertures increased. Consequently, they argued that the overall increase in the hand opening during monocular viewing constitutes a recalibration of safety margins due to the increased perceptual uncertainty, rather than an overestimation of object size. Our results support this argument by showing that, while increases in obstacle size were indeed underestimated in monocular reaching (as evident in shallower slopes), safety margins increased (as indicated by larger intercepts).

While findings on the hand apertures seem quite consistent across studies, the effects on other components of the hand trajectory have been less robust. That is, studies find conflicting results on whether monocular viewing also disrupts the transport component of the grasp, i.e., the part of the movement where the hand approaches the object to get into grasping range. While some argued that hand transport is unaffected by the removal of binocular information^12^, others reported prolonged movement times and decreased accuracy in approach parameters^13,23,26^ suggesting that the reaching movement also critically depends on the availability of binocular information. Here, we found that the effects of sensitivity to obstacle height and safety margins on hand transport in our reaching task reflected exactly those effects previously reported for the maximum grip aperture in grasping tasks. This congruency in action adaptation in response to increased perceptual uncertainty emphasises the role of task relevance. While object size is largely irrelevant for the reaching component of the trajectory during grasping, our variations in obstacle height (which were essential for obstacle avoidance) strongly and consistently influenced behaviour.

Previous research has shown that potential consequences of actions have an effect on the magnitude of the avoidance response^18,27,28^, but in all these studies the manipulation of consequences resulted from a change in the appearance of the obstacles. Here, we were able to separate the effects of consequences and appearance and found that both *independently* influenced the safety margins. This independence of the two effects would not necessarily have been expected, because when there are no consequences to the inaccurate execution of movements, perceptual uncertainty might matter less, or not at all. Our results fit with those from studies on sensorimotor decision-making where, in the absence of penalties, increased motor uncertainty or decreased spatial distances between reward and penalty region did not influence the endpoints of pointing movements, whereas in conditions with penalties they did^29^. Our results show that perceptual uncertainty does not become irrelevant to actions in the absence of error-related costs, suggesting that there are two separate processes influencing the safety margin: One that adjusts movements due to purely perceptual criteria and one that adjusts for other—higher level—task relevant factors, that we have described as the consequences of actions. The perceptual process is oblivious to the differences between mirrored and real presentations because perceptually they are identical. This is supported in our results by the similar slopes for the *mirror* and *real* conditions. The second process only evaluates the higher-level aspects.

With respect to sensorimotor decision making^6^, our results show that even in a task without a speed-accuracy trade-off, feedback, explicit payoff values or a specific instruction to maximize gains, participants still seem to strive for behaviour that balances safety concerns and efforts. Of course, we cannot make any specific predictions about the optimality of these movements, but we can make a couple of related observations. Since participants performed the task without tight time constraints (see Methods for details), it would have been possible for participants to choose fixed, broad safety margins, across the whole experiment, that were large enough for the effects of perceptual uncertainty on the estimation of (real) obstacle height to be safely ignored. They did not. Similarly, in the mirror condition, participants could have ignored differences in height between the obstacles because there would have been no consequences if they had been moving too low. However, this is also not what their movements showed. They seemed to avoid making unnecessarily high movements, which would require more effort, by only making the adjustments necessary to compensate for differences in sensitivity or inferred costs. It is difficult to say to what degree the adjustment due to differences in costs is under deliberate control, but the persistent difference between trajectories in the monocular and binocular viewing conditions independent of the costs suggests an automatic behaviour. Even if participants in the mirror condition somehow felt compelled to mimic passing real obstacles, it would be very unlikely that they could deliberately have simulated the differences between monocular or binocular viewing.

### Implications for VR/AR

Our results showed that, first, avoidance behaviour differed in natural and virtual environments and second, that this difference was not alone the result of impoverished visual stimulation. From this it follows that the behavioural differences between natural and virtual environments will not simply disappear with improvements in display hard- and software.

The concept of (virtual) presence, i.e., the “experience of being there”, has been suggested as a metric for the quality of virtual environments although there seems little agreement about how virtual presence should be operationalised and measured^30,31^. Meehan et al.^32^ explored physiological parameters as operationalisations of virtual presence. They investigated whether stressful virtual environments can induce changes in physiological parameters and found that changes in heart rate satisfied their requirements for a measure of presence. Here, we used the correspondence of actions (obstacle-avoidance behaviour) in natural and virtual environments as a measure of how well a virtual environment could potentially simulate a natural environment. While the appropriateness of a metric for presence will likely be task-dependent, for many practical applications action-correspondence might be a relatively easily attainable metric with high ecological validity.

While the majority of behavioural research into VR/AR has focussed on perceptual aspects of virtual environments, particularly the implementation of naturalistic 3D information, much less is known about the effects of ’virtuality’ itself, i.e., the immaterial nature of environments and objects, on behaviour^1^. The systematic investigation of these effects on behaviour with currently available VR/AR devices is difficult because the effects of impoverished visual stimulation and virtuality are confounded. For example, if we find an increase in safety margins in an obstacle avoidance task when using a VR/AR device, we cannot easily determine if and to what degree this increase was caused by the impoverished visual stimulation and/or by different expectations of consequences due to the virtual nature of the obstacle. Our mirror reflection manipulation was specifically designed to isolate these effects and investigate them under otherwise identical experimental conditions. This comparison of behaviours in real and mirrored environment can be thought of as a ’benchmark’ of how much similarity between actions in natural and simulated environments can possibly be achieved if identical visual information was available in natural and simulated environments. The lower baseline safety margins that we found for the mirrored obstacles compared to the real obstacles indicate that the virtual nature of the obstacles influences movement trajectories *independently* from the effects of perceptual uncertainty. We suggest that the critical aspect that causes this difference in behaviour is the different expectations of error-related costs. In particular, the absence of any direct consequences resulting from a collision with virtual obstacles (when viewing mirror reflections), resulted in riskier behaviour, i.e., smaller baseline safety margins. The issue of expected consequences therefore has to be considered when VR/AR devices are used to study behaviour and—more importantly—when they are used to train or control actions that have to be executed precisely and where the consequences of potential mistakes can be severe, e.g., medical application like robot-assisted surgery^33,34^.

A challenge for the development of VR/AR is whether it is possible to compensate for the absence of direct consequences to movement errors. For example, in the case of our experiment, would it be possible to achieve similar movement trajectories for real and mirrored obstacles, e.g., by simulating the missing haptic feedback in case of a collision with the mirrored obstacle using a force-feedback device or by introducing monetary penalties? A further aspect, which our experiment did not address directly, are the influences of motor uncertainty and biomechanical effort. Both have been shown to shape movements as well^15–17^. This will be especially relevant if users need to move to control virtual representations, e.g., pointers or simulated limbs. In this case, the relation between effort and movement magnitude or direction will differ from their natural relation. Obviously, for many applications of VR/AR exactly this is intended and highly desirable. However, for situations where we want behaviour acquired and trained in VR/AR to transfer in the expected way to their corresponding real-world counterparts, these differences need to be considered. For example, if in an obstacle avoidance task making a large avoidance movement required a smaller increase in effort in VR than in natural environments, then safety margins might be trained to be larger despite similar perceived danger. To sum up, isolating relevant sensory information is only one part of the challenge that VR/AR faces, identifying other task relevant factors like error-related costs or biomechanical effort is also crucial when, for specific purposes, we require similar actions in natural and virtual environments.

In conclusion, our results strikingly demonstrate that even when exact perceptual correspondence between natural and simulated environments is achieved, action correspondence does not necessarily follow due to a disparity in the expected consequence of actions in the two environments.

## Methods

### Setup

**Figure 2:**
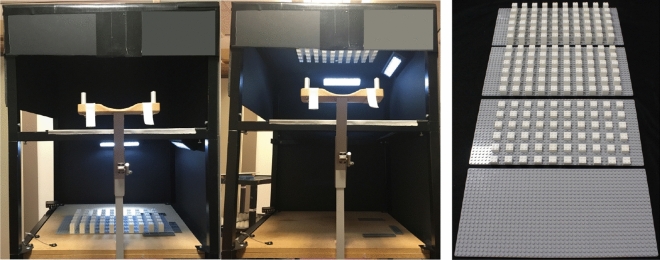
Setup and stimuli. Mirror setup with obstacle placement in the *real* condition (left) and obstacle placement in the *mirror* condition (middle). The mirror located in the centre of the box projects the obstacles so that their mirror images appear in the same location as the obstacles in the *real* condition. Obstacles used in the experiment (right) from bottom to top: base board, one brick, two bricks, three bricks.

We used a custom-made mirror setup (Fig. 2) to present the stimuli. The mirror setup consists of a box (W 56 cm $\times$ D 56 cm $\times$ H 68 cm). The box is separated into a top and bottom part by a semi-transparent mirror (56 cm $\times$ 40 cm) placed in the vertical centre of the box, i.e., the workspace below the mirror in which in the participants’ hand moved had a height of approximately 34 cm. Obstacles were placed below the mirror (real condition, Fig. 2 left) or upside-down above the mirror (mirror condition, Fig. 2 middle). Participants sat in a height-adjustable chair in front of the setup with their head on a chin-rest looking down into the mirror. Regardless of whether the stimuli were placed below or above the mirror, the participants always saw them in the same location and orientation below the mirror. In the real condition, two battery-operated LED lamps were placed above the obstacles but below the mirror illuminating the obstacles from the front and the right, while in the mirror condition the lamps were placed above the mirror illuminating the mirror images of the obstacles from the front and the right. The start position for the participants' index finger was 3 cm in front of the obstacles and the target location, indicated by a yellow circular marker, was located 6 cm beyond the obstacles. The distance from the start position to the target position was 28 cm. Through an opening in the left side of the box, hand movements were measured using an infra-red based Optotrak 3020 system (Northern Digital Incorporation, Waterloo, Ontario, Canada) with a sampling rate of 200 Hz tracking one infra-red light emitting diode that was attached to the nail of the index finger of the right hand. Participants’ vision was occluded using liquid crystal shutter goggles (PLATO Translucent Technologies, Toronto, Ontario^35^). The experiment was programmed in MATLAB (Mathworks, Natick, MA, USA) using the Optotrak Toolbox^36^.

### Stimuli

We used obstacles of four different heights (Table 1 and Fig. 2, right). Based on pilot experiment, we chose the height of the highest obstacle so that the height of the avoidance movements would not be limited by the height of the workspace (34 cm). The obstacles were constructed from LEGO material. The lowest obstacle consisted of an empty grey LEGO base board (W 383 mm $\times$ D 190 mm $\times$ H 3.4 mm). The studs on the base board had a height of 1.8 mm. The plane part of the base board had a height of 1.6 mm. The other obstacles were created by erecting six rows of 10 LEGO towers consisting of one, two, or three white square LEGO bricks (W 16 mm, D 16 mm, H 11.4 mm), respectively, on top of the base board. The studs on the bricks had a height of 1.8 mm. The bricks without the studs had a height of 9.6 mm. The distance between towers was 16 mm. Overall the area covered by the bricks was W 303 mm $\times$ D 175 mm.

**Table 1:** Heights of the four obstacles.

Obstacle	Height (mm)
Base board	3.4 ($1.6 + 1.8$)
1 Brick	13.0 ($1.6 + 1.8 + 1 \times 9.6$)
2 Bricks	22.6 ($1.6 + 1.8 + 2 \times 9.6$)
3 Bricks	32.2 ($1.6 + 1.8 + 3 \times 9.6$)

The studs on the bricks and the base board had a height of 1.8 mm, the plane part of the base board had a height of 1.6 mm. The bricks without the studs had a height of 9.6 mm.

### Procedure

Ten of the participants started with the *mirror* condition and 11 started with the *real* condition. *Mirror* and *real* conditions were tested in separate sessions (separated by at least one day). Within each condition, participants always started with the *monocular* viewing condition followed by the *binocular* condition. In the *monocular* condition, the participants’ non-dominant eye was occluded with an eye-patch. The shutter goggles were closed between trials when the experimenter positioned the obstacle boards. They opened together with an auditory go-signal at the beginning of each trial. If in a trial participants started before the auditory signal or within 100 ms after the signal, the trial was excluded and repeated at a random position within the experiment. As soon as the participants’ right hand started to move, i.e., the 3D Euclidean distance between the marker and the start position exceeded 25 mm, the glasses closed again. Thus, all movements were performed visually open-loop. The four different obstacle heights were presented in pseudo-randomized order in each condition. There were ten repetitions per obstacle height. In total, each participant performed 160 trials. Before the start of each trial, the experimenter changed the obstacle board placing it either above or below the mirror. The participants were instructed to move their hand over the obstacles presented in front of them and to touch the target patch placed beyond the obstacle board and return to the start position. Participants were told to wait for the auditory signal and start moving their hand as soon as possible. There was no instruction regarding the speed of the movements but the movement should have been finished within 3 s. Trials where the movement duration exceeded 3 s were excluded and repeated. Participants were encouraged to be as accurate as possible in hitting the target and to make natural hand movements, but there was never any feedback regarding their accuracy. Participants were not informed about the different presentation conditions, i.e., *mirror* and *real*, because telling them explicitly about the difference between the *mirror* and *real* conditions could potentially have biased their avoidance behaviour in the hypothesised direction. By not informing participants, we reduced the chances of detecting a difference between these conditions. However, we deliberately did not try to conceal this difference from the participants. There were several ways in which they could have detected the differences between the *mirror* and *real* conditions. Obviously, they could just have found out by trying to touch the obstacles in the *mirror* condition. Although vision was occluded between trials, auditory cues could have indicated whether the experimenter exchanged the stimuli at the top or the bottom of the mirror setup (see Fig. 2). Another difference between the *mirror* and the *real* conditions was the placements of the two lamps which illuminated either the real obstacles or the mirror and hence were placed either below or above the mirror. Since the *mirror* and *real* conditions were tested in different sessions on different days, participants might not have noticed this difference. After the experiment, we asked participants whether they had noticed a difference between the *mirror* and *real* conditions. Of 21 participants, 16 participants reported having noticed the difference, and five reported having not noticed the difference. We decided to present the data of the complete dataset since we think it would be inappropriate to separate the dataset based on the informal interview of the participants after the experiment. Moreover, we cannot be absolutely certain whether the five participants who did not report a difference between the *mirror* and *real* conditions were not aware of the difference between the conditions, or whether they were aware of it but did not perceive a (visual) difference. In the Supplementary Information, we present the mean slopes and intercepts as well as statistical analysis separately for the groups of participants who reported a difference between the *mirror* and *real* conditions and the participants who did not report a difference (Figure S1 and Tables S1-S3 in the Supplementary Information).

### Participants

We collected data from 21 right-handed participants (age range: 19–39 years, mean (SD) age: 26.5 (5.62)). At the beginning of the first experimental session, the participants’ dominant eye was determined. Participants were asked to extend their arms out in front of them and create a triangular opening between their thumbs. With both eyes open, participants were asked to centre this triangular opening on a red light on the wall (distance from participant to the wall was three meters). Then participants were asked to close their left eye. If the object (the red light) stayed centred, their right eye (the one that was open) was determined as the dominant eye, otherwise the left eye was determined as dominant. For 16 participants the right eye was dominant, for five the left eye. Participants were naive as to the purpose of the experiment and were compensated £10 for their time. The experimental procedures used were in accordance with the Ethics Code of the British Psychological Society, the declaration of Helsinki and approved by the Psychology Ethics Committee of the University of Aberdeen (Ethics code: PEC/4250/2019/7). All participants gave written informed consent.

### Data analysis

The 3D position data of the index finger marker was filtered offline using a second-order Butterworth-filter with a low-pass cut-off frequency of 15 Hz. Trials were rejected when the hand movement started before the auditory start signal, the movement was not finished within 3 s, or the marker was not visible for longer durations during the movement resulting in missing data. Of a total of 3360 trials, 32 trials were excluded from the data analysis. For data analysis, we used the trajectories in the vertical direction (z-dimension) and determined for each trajectory the maximum z-value (peak height) reached between the start and end of the reaching movement. Start and end of the movement were determined using a resultant velocity threshold of 0.05 m/s. For each participant and condition, we computed the simple linear regression using a least-squares criterion to predict peak movement height based on obstacle height. The resulting slopes and intercepts were then averaged over participants separately for each condition.

For statistical analysis, movement data were analysed using a 2 $\times$ 2 $\times$ 4 repeated-measures ANOVA with the factors *perceptual uncertainty* (binocular vs monocular), *costs* (mirror vs real), and *obstacle height* (3.4, 13.0, 22.6, and 32.2 mm). Intercepts and slopes were analysed using a 2 $\times$ 2 repeated-measures ANOVA with the factors *perceptual uncertainty* and *costs*. If Mauchly’s Test of Sphericity indicated that the assumption of sphericity had been violated (${p}<.05$), Greenhouse–Geisser corrected values were used (indicated by fractional degrees-of-freedom).

To test whether there was a difference between those participants who started with the *mirror* condition and those who started with the *real* condition, for both intercepts and slopes 2 $\times$ 2 repeated-measures ANOVAs with the within-subject factors *perceptual uncertainty* and *costs* and the between-subject factor *order-of-presentation* were run. There was neither a significant main effect of *order-of-presentation* on intercepts (${F}(1,19)=1.646$, ${p}=.215$, $\eta _p^2=0.080$; all interactions: ${p}\ge .234$, $\eta _p^2\le 0.074$) nor slopes (${F}(1,19)=0.076$, ${p}=.786$, $\eta _p^2=0.004$). For slopes, there was a significant interaction between *costs* and *order-of-presentation* (${F}(1,19)=15.083$, ${p}<.001$, $\eta _p^2=0.443$; all other interactions: ${p}\ge .123$, $\eta _p^2\le 0.121$).

## Supplementary information

Supplementary Information.

## Data Availability

The data are available online from the Open Science Framework: https://osf.io/vskxy/.
