# Supplementary material for: Perceptual uncertainty and action consequences independently affect hand movements in a virtual environment
Source: Sci Rep. 2020 Dec 18;10:22307. doi: 10.1038/s41598-020-78378-z (PMC7749146; doi:10.1038/s41598-020-78378-z)
Supplement: Supplementary file 1 — Supplementary Information. [file 41598_2020_78378_MOESM1_ESM.pdf]

Perceptual uncertainty and action consequences independently affect hand movements in a virtual environment

Martin Giesel[a,*], Anna Nowakowska[a], Julie M. Harris[b], and Constanze Hesse[a]

[a]School of Psychology, University of Aberdeen, Aberdeen, AB24 3FX, UK
[b]School of Psychology and Neuroscience, University of St Andrews, St Andrews, KY16 9JP, UK
[*]martin.giesel@abdn.ac.uk

Supplementary information

Analysis of results by awareness of different conditions

Participants were not informed about the two different presentation conditions, i.e., *real* and *mirror*. After the experiment, we informally asked participants whether they had noticed the difference between the *real* and *mirror* conditions. Of 21 participants, 16 reported that they had noticed the difference between the *mirror* and *real* conditions, five reported that they had not noticed the difference. Here, we present the results separately for these two subsets of participants and compare them to the findings for the complete dataset. Figure S1 and Tables S1 and S2 show the mean slopes and intercepts for the complete dataset (N=21, left column), for the group of participants who reported a difference between the *real* and *mirror* conditions (N=16, middle column), and the group of participants who did not report a difference between these conditions (N=5, right column). The data for the complete dataset is the same as presented in Figure 1D in the main text. The mean slopes and intercepts for the group of participants who reported a difference between the *real* and *mirror* conditions were very similar to those for the complete dataset.

We performed the same statistical analysis of the mean slopes and intercepts, respectively, for the subset of participants who reported a difference between the *real* and *mirror* conditions and compared the results to those for the complete dataset. Table S3 shows the results for the 2 $\times$2 repeated-measures ANOVA for slopes and intercepts, respectively, with the factors *perceptual uncertainty* and *costs* for the two datasets. The pattern of results remained unchanged for the reduced sample of N=16.

Given the small number of participants (N=5) in the group of participants who did not report a difference between the *real* and *mirror* conditions, performing a statistical analysis analogue to the one performed for the other datasets (i.e., 2 $\times$ 2 repeated-measures ANOVA) is inappropriate. The mean slopes and intercepts for this group show a tendency that is consistent with our predictions (Figure S1, right column). Since we hypothesised that the mirror should not affect perceptual uncertainty, we would not expect that the lack of awareness of the difference between the *mirror* and *real* conditions has an effect on the slopes. While the mean slopes for this group were in general steeper than those for the complete dataset, the effect of perceptual uncertainty (monocular and binocular viewing) on the slopes was similar, i.e., the slopes for low uncertainty (binocular viewing, Real & Bin and Mirror & Bin in Figure S1, right column, top row) were roughly twice as steep as the slopes for high uncertainty (monocular viewing, Real & Mon and Mirror & Mon in Figure S1, right column, top row).

We further hypothesized that intercepts would be influenced both by perceptual uncertainty and the expected consequences. So, for participants who did not notice a difference between the *mirror* and *real* conditions, we would expect the intercepts to only be affected by perceptual uncertainty. This would result in a similar pattern of results for the intercepts as for the slopes, i.e., higher intercepts for monocular viewing and lower intercepts for binocular viewing with no differences between the *mirror* and *real* conditions. We clearly see this similarity for binocular viewing (Real & Bin and Mirror & Bin in Figure S1, right column, bottom row). As expected, intercepts for monocular viewing (Real & Mon and Mirror & Mon in Figure S1, right column, bottom row) were higher than those for binocular viewing. However, descriptively the monocular intercepts for *mirror* and *real* do not appear to be identical (Mirror & Mon and Real & Mon), but the number of participants in this group is too small to draw any strong conclusions. Moreover, we cannot be absolutely certain whether the five participants who did not report a difference between the *mirror* and *real* conditions were not aware of the difference between the conditions, or whether they were aware of it but did not perceive a (visual) difference.

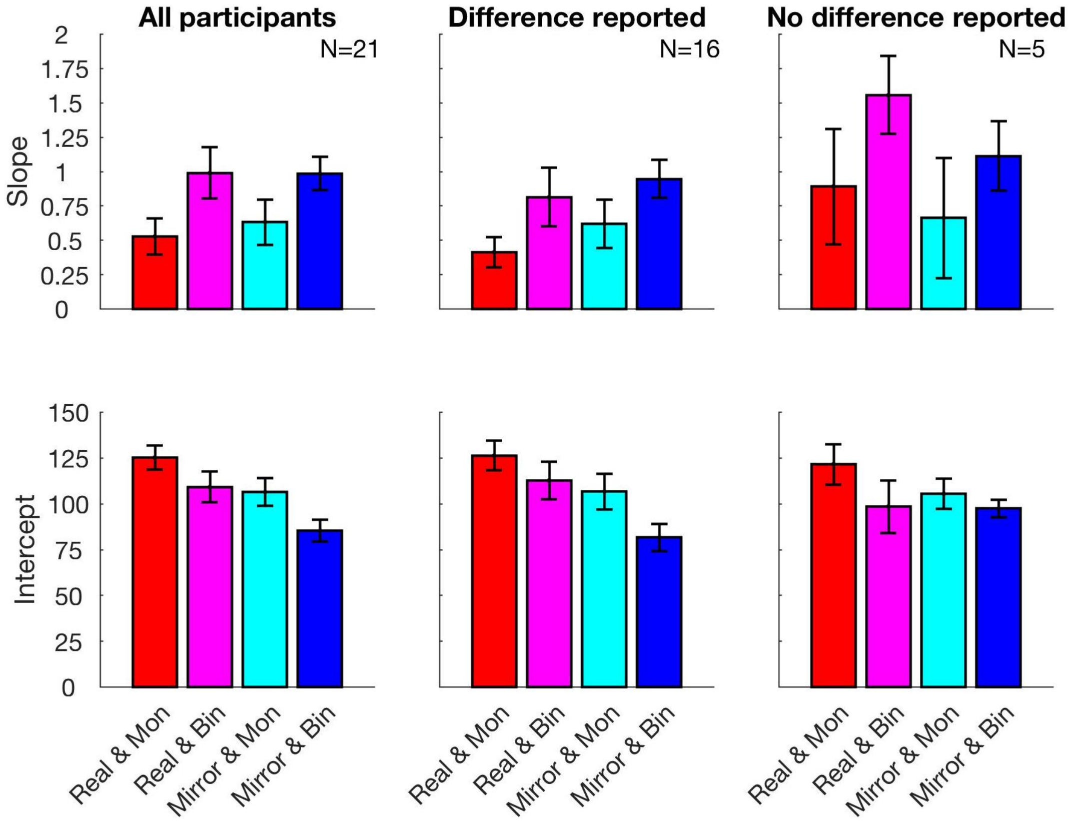

Figure S1. Mean slopes (top row) and intercepts (bottom row) for different groups of participants. (Left column) Data for all participants. These are the same data as depicted in Figure 1D of the main text. (Middle column) Data for participants who reported a difference between the *real* and *mirror* conditions. (Right column) Data for participants who reported no difference between the *real* and *mirror* conditions. Error bars show ± 1 SEM. The label 'Mon' stands for monocular viewing and the label 'Bin' for binocular viewing.

Table S1. Mean and SEM for slopes for all participants (N=21), participants who reported a difference between the *real* and *mirror* conditions (N=16) and participants who did not report a difference (N=5). The label 'Mon' stands for monocular viewing and the label 'Bin' for binocular viewing.

		Real & Mon	Real & Bin	Mirror & Mon	Mirror & Bin
All participants	M	0.5256	0.9912	0.6309	0.9861
	SEM	0.1319	0.1870	0.1634	0.1202
Difference reported	M	0.4114	0.8144	0.6210	0.9461
	SEM	0.1103	0.2140	0.1755	0.1393
No difference reported	M	0.8910	1.5570	0.6624	1.1140
	SEM	0.4191	0.2828	0.4372	0.2545

Table S2. Mean and SEM for intercepts for all participants (N=21), participants who reported a difference between the *real* and *mirror* conditions (N=16) and participants who did not report a difference (N=5). The label 'Mon' stands for monocular viewing and the label 'Bin' for binocular viewing.

		Real & Mon	Real & Bin	Mirror & Mon	Mirror & Bin
All participants	M	125.2	109.3	106.4	85.38
	SEM	6.589	8.424	7.600	5.965
Difference reported	M	126.3	112.6	106.7	81.62
	SEM	8.100	10.18	9.765	7.510
No difference reported	M	121.6	98.42	105.6	97.43
	SEM	10.96	14.43	8.275	4.781

Table S3. Results of 2 × 2 repeated-measures ANOVAs with factors *perceptual uncertainty* and *costs* for slopes and intercepts, respectively, separately for all participants (N=21, same as presented in the main text) and for those participants who reported a difference between the *real* and *mirror* conditions (N=16).

		Factor *perceptual uncertainty*	Factor *costs*	Interaction
Slope	All participants	$F(1,20)=17.744$, $p<.001$, $\eta_p^2=0.470$	$F(1,20)=0.211$, $p=.651$, $\eta_p^2=0.010$	$F(1,20)=0.326$, $p=.574$, $\eta_p^2=0.016$
	Difference reported	$F(1,15)=15.657$, $p=.001$, $\eta_p^2=0.511$	$F(1,15)=1.875$, $p=.191$, $\eta_p^2=0.111$	$F(1,15)=0.107$, $p=.748$, $\eta_p^2=0.007$
Intercept	All participants	$F(1,20)=45.866$, $p<.001$, $\eta_p^2=0.696$	$F(1,20)=31.243$, $p<.001$, $\eta_p^2=0.610$	$F(1,20)=0.443$, $p=.513$, $\eta_p^2=0.022$
	Difference reported	$F(1,15)=49.520$, $p<.001$, $\eta_p^2=0.768$	$F(1,15)=42.845$, $p<.001$, $\eta_p^2=0.741$	$F(1,15)=1.519$, $p=.237$, $\eta_p^2=0.092$
